# Generation of polarization singularities with geometric metasurfaces

**DOI:** 10.1038/s41598-019-56179-3

**Published:** 2019-12-23

**Authors:** Yuchao Zhang, Xiaodong Yang, Jie Gao

**Affiliations:** Department of Mechanical and Aerospace Engineering, Missouri University of Science and Technology, Rolla, MO 65409 USA

**Keywords:** Metamaterials, Nanophotonics and plasmonics

## Abstract

The polarization singularities are directly generated by using plasmonic metasurfaces with the geometric phase profiles designed to form the Poincaré beams. Different morphologies of polarization topological structures of lemon, star, monstar, spiral, dipole and quadrupole are created by the superpositions of Laguerre–Gauss modes with different orders under orthogonal circular or linear polarization basis. The polarization ellipse patterns and topological features of the produced optical vector fields are analyzed to reveal the properties of the polarization singularities of *C*-points and *L*-lines, and the orbital angular momentum states are also measured. The demonstrated polarization singularities generated from the geometric metasurfaces will promise many potential applications related to optical polarization imaging, metrology, optical trapping and quantum information processing.

## Introduction

Optical vortices Polarization singularities carried by optical vector fields have raised great interest due to their unique spatial polarization patterns^1–12^. As one kind of topological defects, polarization singularities represent the points in space that are elliptic dislocations of electromagnetic field and exhibit fine and complicated polarization topological structures, which exist widely in nature such as speckle fields, skylight and oceanography^13–17^. The polarization of electric field is generally described by a polarization ellipse with its orientation angle and ellipticity angle. The space-variant polarization ellipses will form a certain topological structure of the elliptically polarized field, which mainly includes the polarization singularities of *C*-points and *L*-lines. The *C*-points and *L*-lines are located at the positions with the undefined orientation and handedness of the polarization ellipse, where the *C*-points are points of circular polarization surrounded by polarization ellipses with the rotation angles defined by the topological index, and the *L*-lines represent lines of linear polarization separating the left- and right-handed elliptically polarized zones^18–20^. There exist different polarization topological structures for the *C*-point polarization singularities associated with lemon, star, monstar, dipole and quadrupole with the topological index of ±1/2, and spiral with the topological index of +1.

The common methods to generate polarization singularities are based on speckle fields or light propagation through inhomogeneous birefringent media^21–23^. It has been demonstrated that the Poincaré beams which are superimposed by two orthogonally polarized Laguerre–Gauss (LG) modes with different orders can be used to generate polarization singularities^24–28^. However, complicated interferometer setups need to be implemented to combine two or three different optical beams together with the extra diffractive optical elements such as spatial light modulators and Fourier transform lenses. Recently, the geometric metasurfaces fabricated in ultrathin metallic films have been developed to manipulate light phase and polarization^29–34^. Since the geometric phase originates from polarization conversion rather than optical path difference, the geometric metasurfaces have no dispersion and can operate in a broadband spectrum. The geometric metasurfaces have been widely used for realizing the wavefront shaping devices such as flat optical lenses^35–40^, vortex beam converters^41–44^, optical holograms^45–49^, and wave plates^50–53^.

In this paper, the polarization singularities are directly generated by the plasmonic geometric metasurfaces made of V-shaped nanoslit antenna arrays. Based on the design principle of Poincaré beams, six different morphologies of polarization topological structures for the *C*-point polarization singularities associated with lemon, star, monstar, spiral, dipole and quadrupole are created by the superpositions of LG modes with different orders under orthogonal circular or linear polarization basis. With only one single metasurface, the off-axis propagated right- and left-handed circularly polarized (RCP and LCP) LG modes are generated simultaneously and superimposed to prepare the Poincaré beams for tailoring the polarization singularities. The polarization ellipse patterns and topological features of the produced optical vector fields are analyzed to reveal the properties of the polarization singularities of *C*-points and *L*-lines. Furthermore, the orbital angular momentum states are also measured by the optical interference. The demonstrated polarization singularities generated from the geometric metasurfaces will promise many potential applications related to optical polarization imaging, metrology, optical trapping, hybrid entanglement of polarization and OAM, and quantum information processing^54–58^.

## Results

### Design of geometric metasurface

**Figure 1:**
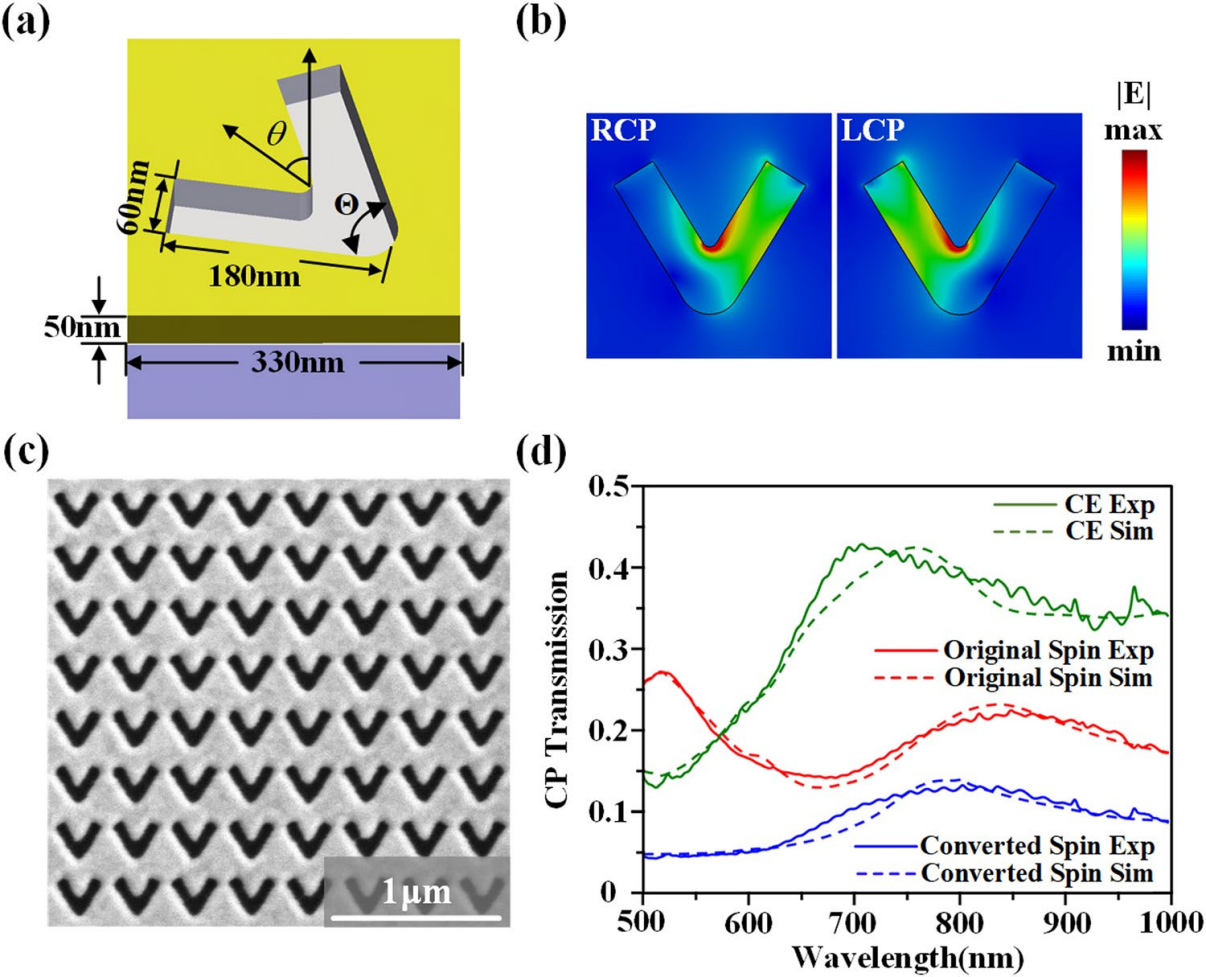
(**a**) Schematic of the V-shape nanoslit at the orientation angle *θ*. (**b**) Simulated electric field |*E*| distributions of V-shape nanoslit under circular polarizations at 633 nm. (**c**) A SEM image of the homogeneous V-shape nanoslit array. (**d**) Simulated and measured transmission spectra under circular polarizations.

The V-shaped nanoslit antenna is adopted to construct the plasmonic geometric metasurface. As shown in Fig. 1a, the V-shape nanoslit is etched in a gold film with thickness of 50 nm on glass substrate using focused ion beam (FIB). The V-shape nanoslit has the width of 60 nm, the arm length of 180 nm, the included angle between two arms Θ of 60°, and the unit cell period of 330 nm. The orientation angle *θ* of the V-shape nanoslit is defined as the angle between the vertical axis and the symmetry axis of the nanoslit. The metasurface contains V-shape nanoslit arrays with the same geometry but different rotation angles. As the original circularly polarized light transmits through the nanoslit unit cell, the transmitted beam consists of the converted spin component with the introduced geometric phase shift and the original spin component without phase shift. For RCP incidence the transmitted LCP component has a geometric phase shift of 2*θ*, while for LCP incidence the transmitted RCP component has a reversed geometric phase shift of −2*θ*^59,60^. Figure 1b shows the simulated electric field |E| distributions at the wavelength of 633 nm, and it indicates strong polarization anisotropy where the intensity is mainly located in the right or left arm for the RCP or LCP component. Figure 1c is the scanning electron microscope (SEM) image of the fabricated homogeneous array of V-shape nanoslits, with the simulated and measured transmission spectra presented in Fig. 1d. The original spin transmission is defined as the power ratio between the transmitted RCP component and the incident RCP beam, while the converted spin transmission is defined as the power ratio between the converted LCP component and the incident RCP beam, and the polarization conversion efficiency (CE) is defined as the power ratio between the converted LCP component and the total transmitted beam. From Fig. 1d, it is observed that the maximum conversion efficiency is around 43% near the wavelength of 700 nm, where the plasmonic resonance occurs.

The design principle of the Poincaré beams can be used to generate the polarization singularities with the desired morphologies of polarization topological structures. A Poincaré beam can be prepared by the superposition of two LG modes with different orders under orthogonal polarization basis as

1 $${\bf{E}}={\psi }_{1}{{\bf{e}}}_{1}+{\psi }_{2}{{\bf{e}}}_{2}$$

where **e**_1,2_ represent two orthogonal polarizations, and the corresponding *Ψ*_1,2_ are the LG modes with different topological charges (TC) of helical phase. **e**_1_ = **e**_*X*_ and **e**_2_ = **e**_*Y*_ under orthogonal linear polarization basis, with **e**_*X*_ and **e**_*Y*_ representing horizontal linear polarization (HLP) and vertical linear polarization (VLP). Under orthogonal circular polarization basis, ${{\bf{e}}}_{1}={{\bf{e}}}_{R}=({{\bf{e}}}_{X}+i{{\bf{e}}}_{Y})/\sqrt{2}$ and ${{\bf{e}}}_{2}={{\bf{e}}}_{L}=({{\bf{e}}}_{X}-i{{\bf{e}}}_{Y})/\sqrt{2}$, with **e**_*R*_ and **e**_*L*_ corresponding to RCP and LCP.

**Figure 2:**
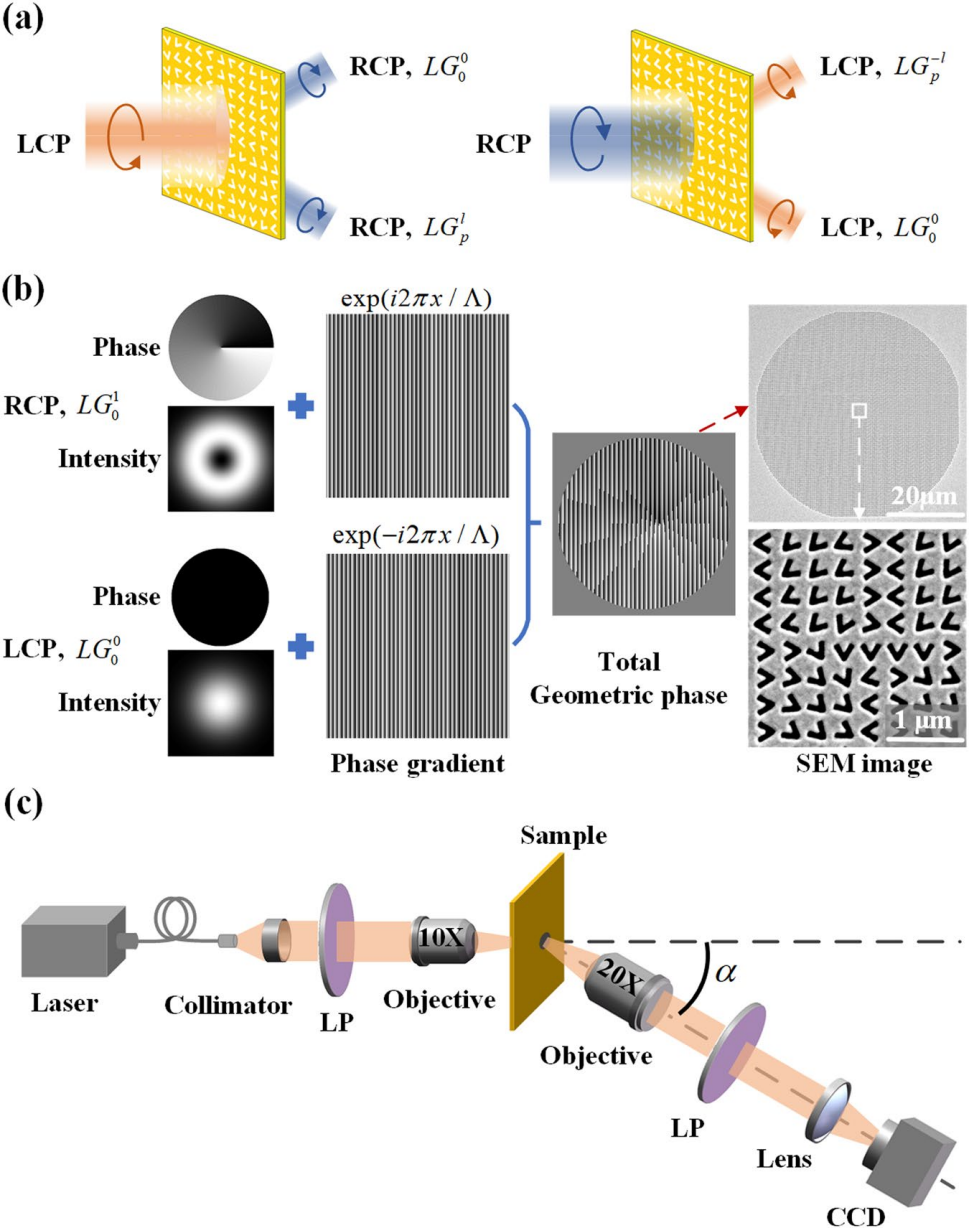
(**a**) The converted RCP and LCP components through the metasurface. (**b**) The design process of the geometric phase profile for generating the lemon morphology, together with the SEM images of total metasurface and its central region. (**c**) Schematic of the experimental setup to characterize the polarization singularity.

The metasurface is designed to generate the optical vector field represented by Eq. (1). In order to separate the converted spin component from the original spin component, the geometric phase profile of the metasurface is imposed with a linear phase gradient. Thus, the converted spin component is deflected by an off-axis angle with respect to the original spin component, so that the incident LCP or RCP beam is converted into two off-axis propagated RCP or LCP beam, as shown in Fig. 2a. As the input beam is linearly polarized, the output converted beam will be the superposition of both the RCP and LCP components. It is noted that even though the output converted beam is circularly polarized, **e**_1,2_ in Eq. (1) can be under linear polarization basis. In this situation, the basis transformation is adopted to transform linear polarization basis into circular polarization basis as ${{\bf{e}}}_{X}=({{\bf{e}}}_{R}+{{\bf{e}}}_{L})/\sqrt{2}$ and ${{\bf{e}}}_{Y}=-\,i({{\bf{e}}}_{R}-{{\bf{e}}}_{L})/\sqrt{2}$. After such transformation, Eq. (1) can be expressed under linear polarization basis as:

2 $$\begin{array}{c}{\bf{E}}={\psi }_{X}{{\bf{e}}}_{X}+{\psi }_{Y}{{\bf{e}}}_{Y}=[{\psi }_{X}({{\bf{e}}}_{R}+{{\bf{e}}}_{L})-i{\psi }_{Y}({{\bf{e}}}_{R}-{{\bf{e}}}_{L})]/\sqrt{2}\\ \,={\psi ^{\prime} }_{R}{{\bf{e}}}_{R}+{\psi ^{\prime} }_{L}{{\bf{e}}}_{L}\end{array}$$

with $${\psi ^{\prime} }_{R}=({\psi }_{X}-i{\psi }_{Y})/\sqrt{2}$$ and $${\psi ^{\prime} }_{L}=({\psi }_{X}+i{\psi }_{Y})/\sqrt{2}$$.

Next, the complex amplitudes ${\psi }_{1,2}$ are determined according to the desired morphology of polarization topological structure, with ${\psi }_{1,2}={\psi }_{R,L}$ under circular polarization basis and ${\psi }_{1,2}={\psi }_{X,Y}$ under linear polarization basis. As listed in Table 1, six different morphologies of polarization topological structures of lemon, star, monstar, spiral, dipole and quadrupole are created, with the first four designed under orthogonal circular polarization basis ${{\bf{e}}}_{R,L}$ and the last two designed under orthogonal linear polarization basis ${{\bf{e}}}_{X,Y}$. The complex amplitude ${\psi }_{2}$ is always the zero-order LG beam as ${\psi }_{2}=L{G}_{0}^{0}=\exp (-\,{r}^{2}/{w}^{2})$, while the complex amplitude ${\psi }_{1}$ has the common form of LG mode as

3 $$L{G}_{p}^{l}={A}_{l,p}{r}^{|l|}{L}_{p}^{|l|}G\exp (il\varphi )$$

where ${A}_{l,p}$ is a normalization constant, *G* is the Gaussian envelope of $G=\exp (-\,{r}^{2}/{w}^{2})$ with *w* = 20 µm in this work, ${L}_{p}^{|l|}$ is associated with the Laguerre polynomial with *p* = 0, and *l* represents the TC of helical phase with azimuthal phase dependency of exp(*ilφ*).

**Table 1:** Different morphologies of polarization topological structures.

Morphology	${{\bf{e}}}_{1,2}$	${\psi }_{1,2}$
Lemon	${{\bf{e}}}_{R,L}$	${\psi }_{1,2}={\psi }_{R,L}$
		${\psi }_{L}=L{G}_{0}^{0}$, ${\psi }_{R}=L{G}_{0}^{1}$
Star	${{\bf{e}}}_{R,L}$	${\psi }_{1,2}={\psi }_{R,L}$
		${\psi }_{L}=L{G}_{0}^{0}$, ${\psi }_{R}=L{G}_{0}^{-1}$
Monstar	${{\bf{e}}}_{R,L}$	${\psi }_{1,2}={\psi }_{R,L}$, *β* = 30°.
		${\psi }_{L}=L{G}_{0}^{0}$, ${\psi }_{R}=cos\beta \cdot L{G}_{0}^{1}-\,\sin \,\beta \cdot L{G}_{0}^{-1}$
Spiral	${{\bf{e}}}_{R,L}$	${\psi }_{1,2}={\psi }_{R,L}$
		${\psi }_{L}=L{G}_{0}^{0}$, ${\psi }_{R}=L{G}_{0}^{2}{e}^{i\pi /2}$
Dipole	${{\bf{e}}}_{X,Y}$	${\psi }_{1,2}={\psi }_{X,Y}$
		${\psi }_{X}=L{G}_{0}^{0}$, ${\psi }_{Y}=L{G}_{0}^{1}$
Quadrupole	${{\bf{e}}}_{X,Y}$	${\psi }_{1,2}={\psi }_{X,Y}$
		${\psi }_{X}=L{G}_{0}^{0}$, ${\psi }_{Y}=L{G}_{0}^{2}$

In Table 1, the parameter *β* is the angle to adjust the intensity ratio between $L{G}_{0}^{1}$ mode and $L{G}_{0}^{-1}$ mode. The complex field of RCP component is ${\psi }_{R}=\,\cos \,\beta \cdot L{G}_{0}^{1}-\,\sin \,\beta \cdot L{G}_{0}^{-1}$, which is determined by the angle *β*. As *β* = 0°, the RCP component is pure $L{G}_{0}^{1}$ mode with topological charge of +1, while as *β* = 90°, the RCP component is pure $L{G}_{0}^{-1}$ mode with topological charge of −1. When 0° < *β* < 90°, the RCP component is the superposition of $L{G}_{0}^{1}$ mode and $L{G}_{0}^{-1}$ mode. In order to generate the monstar polarization singularity, the angle *β* should take the value within 18.43° < *β* < 45°^25^, and here *β* = 30° is adopted.

Based on the obtained complex amplitudes ${\psi }_{1,2}$, the geometric phase profile of metasurface can be calculated. Since the geometric phase is *φ*_*geom*_ for the LCP component but −*φ*_*geom*_ for the RCP component, the phase profiles of the LCP and RCP components are designed separately. First, for the LCP component, the complex amplitude ${\Psi }_{L}={\psi }_{L}$ when ${{\bf{e}}}_{1,2}$ is under circular polarization basis, while ${\Psi }_{L}={\psi ^{\prime} }_{L}$ as defined by Eq. (2) when ${{\bf{e}}}_{1,2}$ is under linear polarization basis. Then a linear phase gradient of $2\pi x/{\rm{\Lambda }}$ (Λ = 1.6 µm) is imposed to the function ${\Psi }_{L}={A}_{L}\exp (i{P}_{L})$. Hence, the converted LCP beam will propagate off-axis at a deflection angle of $\alpha =\arctan (\lambda /{\rm{\Lambda }})={21}^{\circ }$ and the total complex amplitude is ${A}_{L}\exp [i({P}_{L}+2\pi x/{\rm{\Lambda }})]$. Second, the complex amplitude ${\Psi }_{R}={A}_{R}\exp (i{P}_{R})={\psi }_{R}\,{\rm{or}}\,{\psi ^{\prime} }_{R}$ for the RCP component, and the total complex amplitude for the converted RCP beam is ${A}_{R}\exp [-\,i({P}_{R}+2\pi x/{\rm{\Lambda }})]$. Finally, the total geometric phase profile *φ*_*geom*_(*x*, *y*) is obtained by superimposing the complex amplitudes of both RCP and LCP components and then calculating the argument of the summation complex function^60,61^, which can be expressed as

4 $${\Psi }_{L}={A}_{L}\exp (i{P}_{L})={\psi }_{L}\,{\rm{or}}\,{\psi ^{\prime} }_{L}.$$

5 $${\Psi }_{R}={A}_{R}\exp (i{P}_{R})={\psi }_{R}\,{\rm{or}}\,{\psi ^{\prime} }_{R}.$$

6 $${\varphi }_{geom}(x,y)=\text{arg}\{{A}_{R}\exp [-i({P}_{R}+\frac{2\pi x}{{\rm{\Lambda }}})]+{A}_{L}\exp [i({P}_{L}+\frac{2\pi x}{{\rm{\Lambda }}})]\}$$

where ${\psi }_{L,R}\,{\rm{or}}\,{\psi ^{\prime} }_{L,R}$ are selected based on the polarization basis of ${{\bf{e}}}_{1,2}$ being ${{\bf{e}}}_{R,L}$ or ${{\bf{e}}}_{X,Y}$. Figure 2b shows the design process of the geometric phase profile encoded on the metasurface to prepare the Poincaré beam for generating the lemon morphology of polarization topological structure. The experimental setup is shown in Fig. 2c with the generated Poincaré beam propagated at a deflected angle of $\alpha =21^\circ$.

### Characterization of polarization singularities

**Figure 3:**
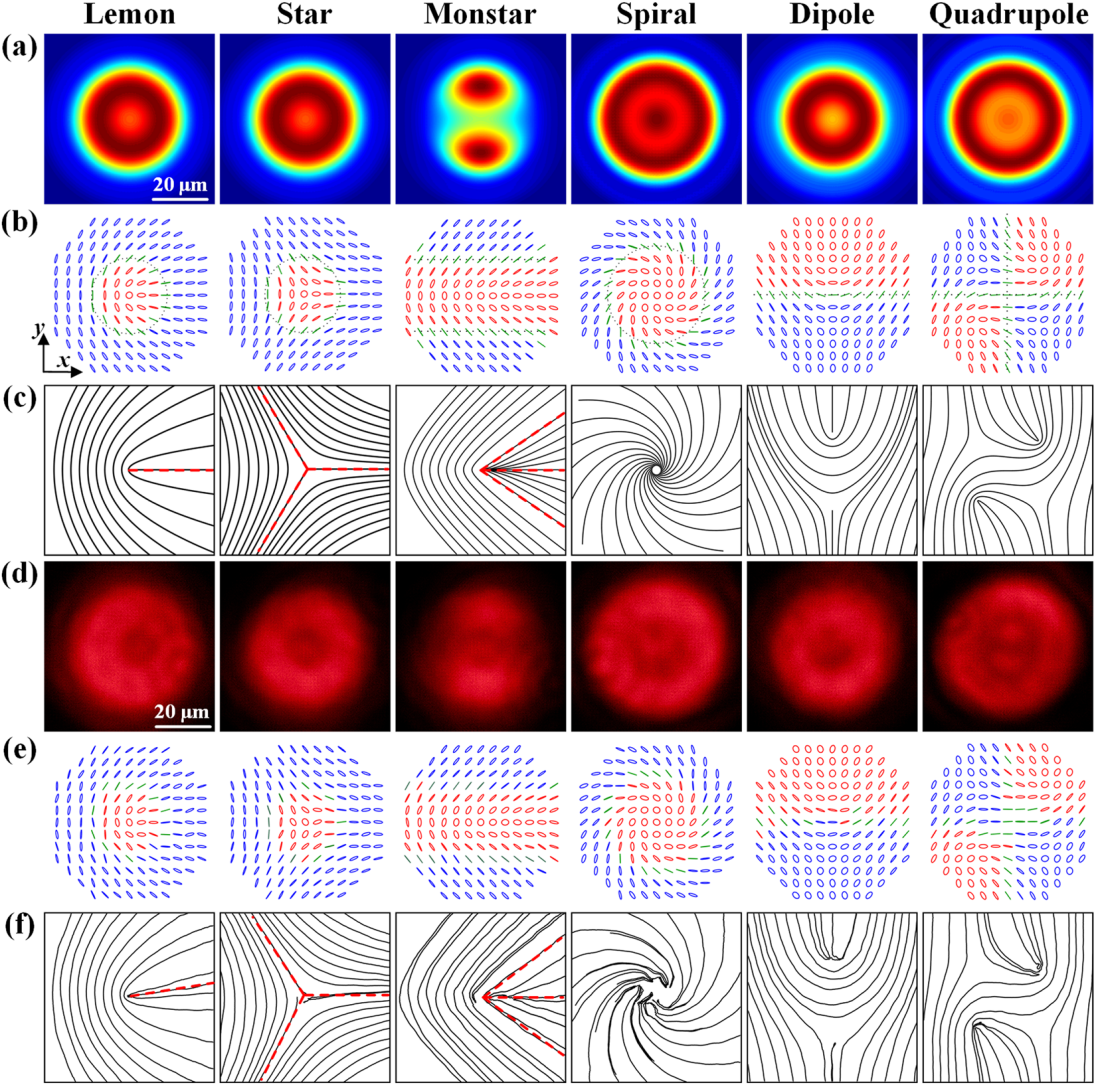
(**a**) Simulated intensity distributions, (**b**) polarization ellipse patterns, and (**c**) polarization topological structures of six different morphologies. (**d**–**f**) Experimental results corresponding to (**a**–**c**). The left-handed polarized ellipses are marked by red color, and the right-handed polarized ellipses are marked by blue color, and the linear polarization states are marked by green color. The *L*-lines are marked as black dotted lines, and the radial polarization lines are marked as red dashed lines.

Figure 3 shows the simulated and experimental results of the six different morphologies of polarization topological structures for the *C*-point polarization singularities associated with lemon, star, monstar, spiral, dipole and quadrupole. Figure 3a displays the simulated intensity distributions for the produced Poincaré beams. Figure 3b plots the polarization ellipse patterns calculated from the Stoke parameters. Figure 3c shows the corresponding polarization topological structures, which is plotted by connecting the semimajor axes of the polarization ellipses. Figure 3d–f are the corresponding measured results of intensity distributions, polarization ellipse patterns and topological structures. The Stokes parameters of *S*_0_, *S*_1_, *S*_2_ and *S*_3_ are obtained by using a quarter-wave plate and a linear polarizer in order to determine the spatially variant polarization distributions^62^. The optical intensities of *I*_0_, *I*_1_ and *I*_2_ are measured by removing the quarter-wave plate and rotating the transmission axis of the linear polarizer into the angles of 0°, 45° and 90°, and the optical intensity of *I*_3_ is measured by reinserting the quarter-wave plate and rotating the fast axis of the quarter-wave plate into 90° and the transmission axis of the linear polarizer into 45°. Then the Stokes parameters can be obtained by the following equations:

7 $$\begin{array}{c}{S}_{0}={I}_{0}+{I}_{2}\\ {S}_{1}={I}_{0}-{I}_{2}\\ {S}_{2}=2{I}_{1}-{I}_{0}-{I}_{2}\\ {S}_{3}=2{I}_{3}-{I}_{0}-{I}_{2}\end{array}$$

After obtaining the Stokes parameters, the azimuth angle *ϕ* and the ellipticity *ε* of the polarization ellipse can be calculated as *ϕ* = 1/2∙arctan(*S*_2_*/S*_1_) and $\varepsilon =\,\tan (1/2\cdot \arctan ({S}_{3}/\sqrt{{S}_{1}^{2}+{S}_{2}^{2}}))$, where *ϕ* is the rotation angle of the semimajor axis of the ellipse. The polarization ellipse is also characterized by the polar angle *χ*, which is related to the ellipticity *ε* = *b*/*a* with tan(π/4 − χ) = *ε*, where *a* and *b* are the semimajor and semiminor axes of the ellipse. The values of the azimuth angle *ϕ* and the polar angle *χ* can be calculated by^24^

8 $$\begin{array}{c}\phi =({P}_{R}-{P}_{L})/2\\ \chi =\arctan \frac{{A}_{L}}{{A}_{R}}\end{array}$$

where *P*_*L*,*R*_ and *A*_*L*,*R*_ are defined in Eqs. (4) and (5), and *χ* takes the value between 0 and π/2 with 0 ≤ *χ* < π/4, *χ* = π/4 and π/4 < *χ* ≤ π/2 for the polarization states of LCP, LP and RCP, respectively. According to Eq. (8), the handedness of polarization ellipse is left-handed when *A*_*L*_ > *A*_*R*_ and right-handed when *A*_*L*_ < *A*_*R*_, but the handedness is undefined for linear polarization state with *A*_*L*_ = *A*_*R*_. It is also noted that the azimuth angle *ϕ* is half of the phase difference between the RCP and LCP components. The *C*-point is defined as the isolated points with circular polarization state, and the surrounding polarization ellipses rotate about the *C*-point. Around the circuit enclosing the *C*-point, the azimuth angle *ϕ* of the ellipse turns through an angle Δ = ∫*dϕ* = ±π or ±2π which corresponds to π-symmetric or 2π-symmetric fields with the topological index *I* = Δ/2π = ±1/2 or ±1^18^, representing the half- or integer-index topologies for the *C*-point polarization singularities^20^. With the undefined handedness of polarization ellipse, the *L*-line polarization singularity is located at the curve along which the polarization state is LP with *A*_*L*_ = *A*_*R*_. As shown in Fig. 3b, the *L*-lines represents the borders separating the left- and right-handed polarized areas, which are marked as black dotted lines. Furthermore, the morphologies of polarization topological structures can also be characterized by a special line called the radial polarization line at which the semimajor axis orientation of polarization ellipse is radial, which is marked as red dashed line in Fig. 3.

The lemon morphological structure is generated from the Poincaré beam with the superposition of RCP $L{G}_{0}^{1}$ mode and LCP $L{G}_{0}^{0}$ mode. Since the RCP $L{G}_{0}^{1}$ mode has zero intensity at the beam center *r* = 0, the polarization state at the beam center is completely LCP where the *C*-point is located. The azimuth angle *ϕ*(*r,φ*) for lemon is calculated as $\phi (r,\varphi )=[{\rm{\arg }}(L{G}_{0}^{1})-\text{arg}(L{G}_{0}^{0})]/2=\varphi /2$. The polarization ellipses rotate counterclockwise around the *C*-point and the total rotation angle Δ = +π, so that the lemon is a half-index singularity with the topological index *I* = +1/2. As *r* increases, the intensity of LCP $L{G}_{0}^{0}$ component decreases while the intensity of RCP $L{G}_{0}^{1}$ component increases, so the polarization ellipticity decreases but the handedness remains left-handed. At $r={r}_{0}=w/\sqrt{2}\approx 14\,\mu {\rm{m}}$, the $L{G}_{0}^{1}$ and $L{G}_{0}^{0}$ components have the equal intensity and the polarization state is LP. As *r* > *r*_0_ the intensity of RCP component becomes larger than that of LCP component, so the polarization ellipticity decreases and the handedness becomes right-handed. From the polarization ellipse patterns of Fig. 3b, it can be seen that the *L*-line is located at the circle *r* = *r*_0_ where the polarization state is LP marked by green color, while inside the circle *r* < *r*_0_ the polarization is left-handed marked by red color and outside the circle *r* > *r*_0_ the polarization is right-handed marked by blue color. Besides, there is only one radial polarization line located at *ϕ*(*r*, *φ*) = 0 for lemon structure, which is marked as red dashed line.

The star structure is formed by the superposition of RCP $L{G}_{0}^{-1}$ mode and LCP $L{G}_{0}^{0}$ mode. Same as lemon, the *C*-point of star is located at the beam center *r* = 0 with pure LCP state. The azimuth angle for star is $\phi (r,\varphi )=[\text{arg}(L{G}_{0}^{-1})-\text{arg}(L{G}_{0}^{0})]/2=-\,\varphi /2$, and the polarization ellipses rotate clockwise around the *C*-point with the total rotation angle Δ = −π, so the star is a half-index singularity with the topological index *I* = −1/2. As *r* increases, the polarization state changes from pure LCP to left-handed elliptically polarized inside the circle *r* < *r*_0_ to LP at the circle *r* = *r*_0_ ≈ 14 µm where the *L*-line is located, and becomes right-handed elliptically polarized outside the circle *r* > *r*_0_. The star structure has three radial polarization lines located at *ϕ*(*r,φ*) = 0, 2π/3 and 4π/3, which are marked as red dashed lines. The monstar structure is constructed from the superposition of LCP $L{G}_{0}^{0}$ mode and RCP mode of $L{G}_{0}^{1}\sqrt{3}/2-L{G}_{0}^{-1}/2$. Similar to lemon, the polarization ellipses rotate counterclockwise around the *C*-point at the beam center with Δ = +π and the topological index *I* = +1/2. The *L*-lines are located at two horizontal lines of $y=\pm \,14\,\mu {\rm{m}}$, and the polarization state is left-handed elliptically polarized between these two lines, but right-handed elliptically polarized outsides these two lines. There are three radial polarization lines located around *ϕ*(*r,φ*) = 0, ±34.3°. The spiral structure is formed by the superposition of RCP mode of $L{G}_{0}^{2}\exp (i\pi /2)$ and LCP $L{G}_{0}^{0}$ mode. The *C*-point of spiral is also located at the beam center with pure LCP state. The azimuth angle *ϕ*(*r*, *φ*) = *φ* + π/4 and the polarization ellipses rotate counterclockwise around the *C*-point with Δ = +2π, so that the spiral is an integer-index singularity with the topological index of +1. As *r* increases, the polarization state changes from pure LCP to left-handed elliptically polarized with the reduced ellipticity in the zone *r* < *r*_1_. At the circle $r={r}_{1}=w/\sqrt[4]{2}\approx 17\,\mu {\rm{m}}$, the $L{G}_{0}^{2}$ and $L{G}_{0}^{0}$ modes have the equal intensity and the polarization state is LP where the *L*-line is located. In the zone *r* > *r*_1_, the polarization state becomes right-handed elliptically polarized.

The dipole and quadrupole morphological structures are formed by the superposition of two LG modes under orthogonal linear polarization basis. The dipole structure is constructed by the superposition of HLP $L{G}_{0}^{0}$ mode and VLP $L{G}_{0}^{1}$ mode. The *L*-line is located at the horizontal axis *y* = 0, where ${A}_{L}=|{\psi ^{\prime} }_{L}|$ and ${A}_{R}=|{\psi ^{\prime} }_{R}|$ have the equal intensity, and the polarization state is left-handed elliptically polarized above the *L*-line and right-handed elliptically polarized below the *L*-line. Along the *L*-line at *y* = 0, the intensity of VLP $L{G}_{0}^{1}$ component is zero so that the polarization state is pure HLP. As *x* varies along the positive *x*-axis, the amplitude of VLP $L{G}_{0}^{1}$ component increases, so that the combined LP rotates counterclockwise. And as *x* changes along the negative *x*-axis, the combined LP rotates clockwise. There are two *C*-points in the half-index dipole structure, one is located at (*x* = 0, *y* = +14 µm) with pure LCP state and the topological index *I* = +1/2, while the other is located at (*x* = 0, *y* = −14 µm) with pure RCP state and the topological index *I* = −1/2. The quadrupole structure is formed by the superposition of HLP $L{G}_{0}^{0}$ mode and VLP $L{G}_{0}^{2}$ mode. The *L*-lines with LP state are located at the axes *x* = 0 and *y* = 0, where ${A}_{L}=|{\psi ^{\prime} }_{L}|$ and ${A}_{R}=|{\psi ^{\prime} }_{R}|$ have the equal intensity. The polarization state is left-handed elliptically polarized in the zones (*x* > 0, *y* > 0) and (*x* < 0, *y* < 0), and right-handed elliptically polarized in the zones (*x* > 0, *y* < 0) and (*x* < 0, *y* > 0). At the center point (*x* = 0, *y* = 0), the polarization state is pure HLP. As *x* varies along positive or negative *x*-axis, the amplitude of VLP $L{G}_{0}^{2}$ component increases, so that the combined LP rotates counterclockwise. And as *y* changes along positive or negative *y*-axis, the amplitude of VLP $L{G}_{0}^{2}$ component decreases, so the combined LP rotates clockwise. There are four *C*-points in the half-index quadrupole structure, two of them are located at (*x* = ±12 µm, *y* = ±12 µm) with pure LCP state and the topological index *I* = +1/2, while the other two are located at (*x* = ± 12 µm, *y* = ∓12 µm) with pure RCP state and the topological index *I* = −1/2.

**Figure 4:**
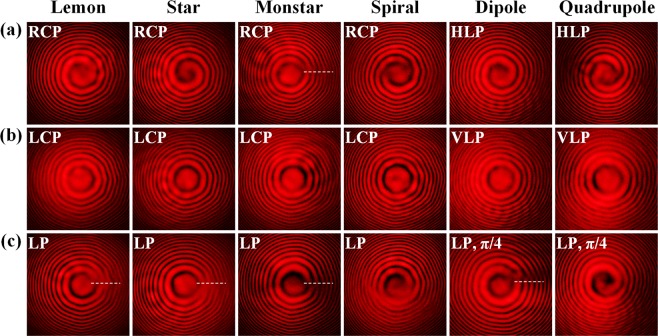
Measured interference patterns for the Poincaré beams interfered with reference spherical waves under different polarization states of (**a**) ${{\bf{e}}}_{1}$ (RCP or HLP), (**b**) ${{\bf{e}}}_{2}$ (LCP or VLP), and (**c**) the combination of ${{\bf{e}}}_{1}$ and ${{\bf{e}}}_{2}$. The white dashed line shows the cutline of spiral discontinuity, indicating the fractional-order TC.

Finally, the OAM states of the produced optical vector fields are also measured by the optical interference, where the Poincaré beams are interfered with a reference spherical wave under different polarization states and the results are shown in Fig. 4. The Poincaré beam is composed by two polarization components under orthogonal circular or linear polarization bases of ${{\bf{e}}}_{1,2}={{\bf{e}}}_{R,L}$ or ${{\bf{e}}}_{X,Y}$ and each component is a LG mode with a specific TC of helical phase, thus both the componential OAM and total OAM are measured. For the componential OAM analysis, the polarization state of reference wave is chosen as either ${{\bf{e}}}_{1}$ (Fig. 4a) or ${{\bf{e}}}_{2}$ (Fig. 4b), so that only the component with the same polarization state as the reference wave is interfered to give the OAM information of that component. For the total OAM analysis, the polarization state of reference wave is set as the combination of ${{\bf{e}}}_{1}$ and ${{\bf{e}}}_{2}$ (Fig. 4c), so that all the components are interfered to show the total OAM information of the optical vector field. And the combination of ${{\bf{e}}}_{1}$ and ${{\bf{e}}}_{2}$ is LP for ${{\bf{e}}}_{1,2}={{\bf{e}}}_{R,L}$ and LP with polarization orientation angle of π/4 for ${{\bf{e}}}_{1,2}={{\bf{e}}}_{X,Y}$. When the reference spherical wave has the polarization state of ${{\bf{e}}}_{1}$, the interference patterns show one counterclockwise-rotated spiral arm for lemon and dipole, one clockwise-rotated spiral arm for star, and two counterclockwise-rotated spiral arms for spiral and quadrupole, indicating their TCs of +1, −1 and +2, respectively. Since the ${{\bf{e}}}_{1}$ component of monstar structure is the superposition of two LG modes with different amplitudes and TCs of +1 and −1, the interference pattern shows a fractional-order TC with a cutline of spiral discontinuity near the beam center, which is marked by the white dashed line in Fig. 4a. For the reference spherical wave with the polarization state of ${{\bf{e}}}_{2}$, all the interference patterns contain no spiral arm as shown in Fig. 4b, because all the ${{\bf{e}}}_{2}$ components are zero-order LG modes with TCs of 0. The total OAM information is given by the interference patterns in Fig. 4c, showing fractional-order TCs for lemon, star, monstar and dipole having a cutline of spiral discontinuity near the beam center, as well as one counterclockwise-rotated spiral arm for spiral and quadrupole with their TCs of +1. It is indicated that the total OAM value of the optical vector field takes the mean value of the TCs of both ${{\bf{e}}}_{1}$ and ${{\bf{e}}}_{2}$ components.

It should be noted that in the current design only the geometric phase of metasurface is employed to generate the phase holograms for the polarization singularities, so that the amplitude information is dropped in Eq. (6). In the design, two polarization singular beams are generated by the metasurface at two deflection angles, so that the efficiency for generating single polarization singular beam is half of the conversion efficiency of the metasurface, which is around 3% at the wavelength of 633 nm. In addition, the noise is introduced due to the dropping of the amplitude of hologram. To further increase the efficiency and reduce the noise, the method described in Ref. ^63^ could be adopted to directly generate the vector beam fields with the designed elliptical polarization distributions.

## Discussion

In summary, the polarization singularities are directly generated with plasmonic geometric metasurfaces via the designed Poincaré beams. Various morphological structures of lemon, star, monstar, spiral, dipole and quadrupole are created by the superpositions of Laguerre–Gauss modes with different orders under orthogonal circular or linear polarization basis. The polarization ellipse patterns and polarization topological structures are studied to understand the properties of the polarization singularities of *C*-points and *L*-lines, which are located at the positions with undefined orientation and handedness of the polarization ellipse, respectively. Furthermore, the componential OAM and total OAM of the produced optical vector field are also analyzed with the interference patterns, indicating that the total OAM takes the mean value of the two componential OAMs. The demonstrated polarization singularities generated from the metasurfaces will advance many related applications in optical polarization imaging, metrology, optical trapping, optical communication and quantum optics.

## Methods

### Simulations

The CST Studio Suite software is employed to simulate the electric field distributions and transmission spectra in Fig. 1 with periodic boundary conditions in the unit cell. The simulated intensity distributions and polarization ellipse patterns in Fig. 3 are obtained from the Fresnel-Kirchhoff diffraction integral^64^ and the Stokes parameters.

### Sample fabrication

Gold film with thickness of 50 nm is deposited on glass slide with electron-beam evaporation. The V-shaped nanoslits are milled with focused ion beam (30 kV, 9.7 pA). The metasurface contains 150 × 150 unit cells with V-shaped nanoslits at the designed orientation angles.

### Optical characterization

The transmission spectra in Fig. 1c are measured with a white light source and a spectrometer. The circularly polarized light is obtained through a linear polarizer and a quarter-wave plate. In Fig. 2c, the optical beam from a HeNe laser at the wavelength of 633 nm is focused onto the metasurface sample through a 10× objective lens. Then the generated optical vector field is collected by a 20× objective lens at the deflection angle of *α* and imaged by a microscope system with a CCD camera.
